# Harmonic focus in total thyroidectomy plus level III-IV and VI dissection: a prospective randomized study

**DOI:** 10.1186/1477-7819-9-141

**Published:** 2011-10-31

**Authors:** Qingqing He, Dayong Zhuang, Luming Zheng, Peng Zhou, Jixin Chai, Zhen Lv

**Affiliations:** 1 Department of Thyroid and Breast Surgery, Jinan Military General Hospital, Jinan 250031, the People's Republic of China

**Keywords:** papillary thyroid microcarcinoma, thyroid surgery, Harmonic Focus, total thyroidectomy, haemostasis

## Abstract

The aim of this study was to compare operating time, postoperative outcomes, and surgical complications of total thyroidectomy plus level III-IV and VI dissection between the no-tie technique using the Harmonic Focus and classic suture ligation for hemostasis. Fifty-four patients underwent total thyroidectomy plus level III-IV and VI dissection by classic suture ligation and 51 patients by the Harmonic Focus. There was obvious distinction as to the operating time between the Focus and classic group (102.8 and 150.1 minutes, respectively, *P *< 0.05). Drainage volume (202.7 ± 187.0 mL vs 299.7 ± 201.4 mL, *P *< 0.05) were significantly lower in the Focus group. Transient hypoparathyroidism had no statistically significant difference between the groups (17.6% vs 18.5%, *P *> 0 .05). No patient experienced nerve injury or permanent hypocalcemia. The use of Harmonic Focus for the control of thyroid vessels during thyroid surgery is reliable and safe. The device can offer extraordinary capabilities for delicate tissue grasping and dissection.

## Background

In early reports, the harmonic scalpel demonstrated some advantages over conventional techniques, particularly in terms of operating time and intraoperative bleeding. The main advantages of ultrasonic coagulating/dissecting systems compared with a standard electrosurgical device are manifested by minimal lateral thermal tissue damage, less smoke formation, no neuromuscular stimulation, and no electrical energy through the patient[1,2]. This new technology is extensively used in laparoscopic surgery and several fields of minimally invasive surgery [3-5]. Harmonic Focus has been developed to which allows for precise and simultaneous cutting as well as hemostasis with minimal damage to surrounding tissues. Safe thyroid surgery requires meticulous hemostasis. Hemostasis in thyroid surgery is achieved by means of the conventional clamp-and-tie technique, diathermy, hemostatic clips, and, recently, the Harmonic Focus.

The aim of this prospective study was to assess the operating time, overall drainage volume, and surgical complications such as hypocalcemia, laryngeal nerve palsy in thyroid surgery with the use of the Harmonic Focus.

## Methods

### Patient Eligibility and Study Design

**Figure 1:**
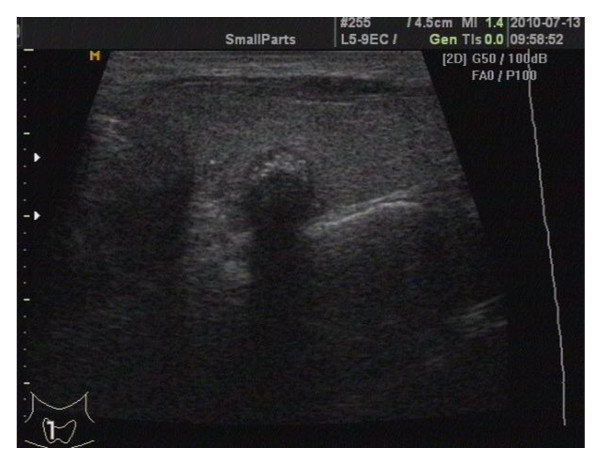
**Thyroid microcarcinoma was discovered by screening with ultrasonography**.

The study involved 13 men and 92 women from January 2010 to September 2010. One hundred and five consecutive patients were preoperatively diagnosed as papillary thyroid microcarcinoma following typically ultrasound identification of nodules and ultrasound guided fine needle aspiration cytology (Figure 1). Exclusion criteria included previous neck operation, a history of neck irradiation. Patients with clinically positive nodes or functional neck dissection were excluded in this study. When level III-IV lymph nodes were positive on frozen section analysis, the dissection was extended to include level IIa and V. The treatment was conducted in accordance with the hospital's ethical standards as set out by its Ethics Committee for Analysis of Research Projects on Human Experimentation. All patients were blinded to the surgical technique used and signed an informed written consent before enrollment to the trial. This prospective randomized trial study was performed on these 105 consecutive patients given total thyroidectomy plus level III-IV and VI dissection (pretracheal, bilateral paratracheal, and prelaryngeal lymph nodes) [6,7]. Patients were randomly assigned to either the Focus group (in which the operation was performed entirely using the Harmonic Focus and no other hemostatic tool; 51 patients) or the classic group (in which the operation was performed using conventional clamp-and-tie technique and mono-polar electric scalpel; 54 patients). The patients all underwent similar treatment following the same protocol, except for the Harmonic Focus used. Total thyroidectomy and level III-IV and VI dissection were performed by the same experienced thyroid surgical team(six adept surgeons) in all cases. The two groups did not display statistically significant differences in term of age, gender, BMI, and pathology classification (Table 1).

**Table 1:** Clinical data between two groups

Variable	Focus Group (n = 51)	Classic Group (n = 54)	*P *-value
Mean age, y[range]	48(27-76)	46.5(23-73)	> 0.05
Sex, No. M/F	6/45	7/47	> 0.05
Diagnosis, No. of patients	51	54	> 0.05
papillary thyroid microcarcinom Body mass index(BMI)	22.8 ± 1.6	22.6 ± 1.7	> 0.05

A complete preoperative assessment (serum thyrotropin levels, parathyroid hormone, serum Ca and P, nodule size by ultrasonography) was obtained in all patients. Preoperative laryngeal nerve status was determined by indirect laryngoscopy, performed by the same otolaryngologist from the Department of Otolaryngology.

### Surgical Technique

**Figure 2:**
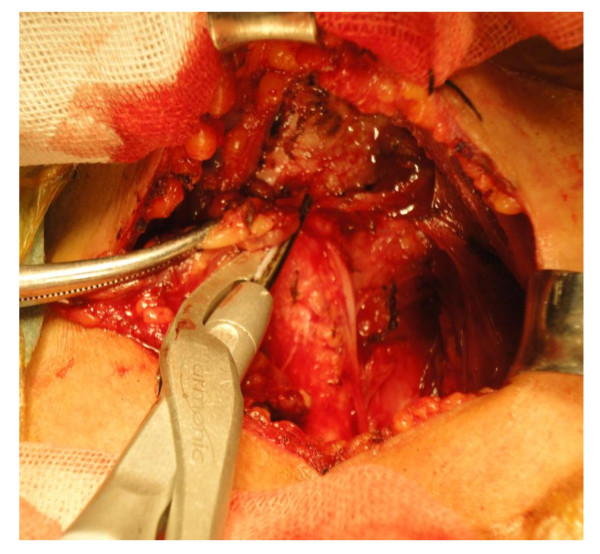
**Paratracheal lymph nodes dissection with the Harmonic Focus, and recurrent laryngeal nerve identified**. (Ethicon Endo-Surgery, Inc, Cincinnati, OH).

Six adept surgeons can use both techniques. Total thyroidectomy plus level III-IV and VI dissection was performed in all patients. Under either block anesthesia cervical plexus or general anesthesia and with endotracheal intubation, the patients were placed on the operating table in the supine position with the neck extended. A 5 cm low-collar incision was made above the sternal notch. After skin incision with the conventional scalpel, flaps were raised using the mono-polar electric scalpel. In the Focus group, we used the Harmonic Focus for vascular control of the thyroid gland (Harmonic Focus, Ethicon Endo-Surgery, Inc, Cincinnati, OH. Figure 2). For better hemostasis, the middle vein, the superior and inferior thyroidal arteries and veins were controlled using level 3. Other small vessels and surrounding connective tissues were controlled using level 5 for easy cutting. For patients in the classic group, mono-polar electric scalpel was used to control the small vessels of the gland and conventional "clip, cut and tie" routines was adapted for the superior and inferior thyroidal arteries, as well as the superior, middle and inferior veins. Total thyroidectomy was first performed. In all patients, we identified recurrent laryngeal nerve routinely. The delicate technique was performed by seeking, identifying and exposing the nerve itself with all branches and following its course with care until it entered larynx. Thyroid gland was examined by frozen section examination during surgery. The tumor size was equal to or less than 10 mm. The parathyroid glands were identified macroscopically, and a meticulous dissection from the thyroid gland was performed. Every effort was made to identify and preserve all parathyroid glands. Parathyroid glands were transplanted in the sternocleidomastoid muscle, if the blood supply to the glands was compromised. Outcomes of the study included operating time, fluid content in the suction balloon (drainage volume), and incidence of complications (rate of hypocalcemia and laryngeal nerve injury). The operative time was measured from initiation of the incision to conclusion of the skin closure. The drains were removed if drainage volume was less than 5 mL in 24 hours.

In all patients, serum parathyroid hormone, serum Ca and P levels were obtained the first postoperative day. Biochemical hypoparathyroidism was defined as serum parathyroid hormone level below 15 ng/L (normal, 15-65 ng/L). Patients with postoperative transient biochemical hypoparathyroidism were giving oral calcium carbonate and calcitriol supplementation, although they showed no clinical symptoms of hypocalcemia. The parathyroid function usually recovers within 3 weeks.

### Statistical Analysis

Statistical analysis of the differences between groups was performed using the 2-sample *t *test and *χ*^2 ^test. Statistical analysis was made by SPSS 13. *P *< 0.05 was considered statistically significant.

## Results

**Figure 3:**
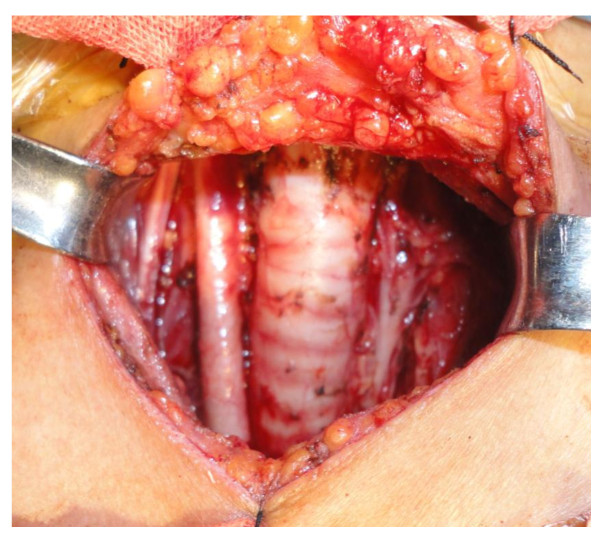
**Total thyroidectomy conbined with level III-IV and VI dissection**.

15 patients(9 cases in the Focus group and 6 in the classic group) with multifocal or bilateral papillary thyroid microcarcinoma received both level III-IV dissection (Figure 3). The final pathology in all the patients was papillary thyroid microcarcinoma. All cases were pN0-1b by TNM-staging [8]. The mean tumor diameter was (6.8 ± 2.8) mm. Of the 105 patients, central cervical lymph node metastases were found in 51 (48.6%) patients. Two cases had 1-2 positive level III or IV lymph nodes on final postoperative paraffin sections, thus they did not receive functional neck dissection again.

Operative and Postoperative parameters for 2 groups were presented in Table 2. Operating time was significantly shorter in Focus group (*P *< 0.05) by allowing a one third time saving vs classic hemostasis. Postoperative transient biochemical hypoparathyroidism occurred more frequently in the classic group than in the Focus group, but no obvious distinction. All patients recovered completely, and no permanent hypoparathyroidism was registered.

**Table 2:** Operative and Postoperative parameters between 2 groups

Variable	Focus Group (n = 51)	Classic Group (n = 54)	*P -value*
Primary tumor size(mm)	6.9 ± 2.5	6.6 ± 2.9	> 0.05
Total operating time(min)	102.8 ± 15.6	150.1 ± 32.9	< 0.05
Time of total			
thyroidectomy(min)	43.5 ± 16.1	69.6 ± 24.6	<0.05
Amount of drainage(ml)	202.7 ± 187.0	299.7 ± 201.4	< 0.05
Time of drainage(d)	5.5 ± 2.5	5.9 ± 2.2	>0.05
Serum PTH (ng/L)			
preoperative	46.90 ± 18.58	43.87 ± 17.21	>0.05
Postoperative	20.6 ± 14.3	19.06 ± 16.88	>0.05
Serum Ca (mmol/L)			
preoperative	2.37 ± 0.14	2.29 ± 0.23	>0.05
postoperative	2.10 ± 0.18	2.07 ± 0.26	>0.05
Serum P (mmol/L)			
preoperative	1.35 ± 0.32	1.38 ± 0.37	>0.05
postoperative	1.44 ± 0.27	1.47 ± 0.29	>0.05
Hypoparathyroidism	9	10	>0.05.
Postoperative			
hospitalization (d)	5.8 ± 1.35	6.7 ± 1.86	<0.05
Parathyroid gland			
autotransplantation(n)	14	16	>0.05.
RLN paralysis			
Temporary(n)	1	0	<0.05
Permanent	0	0	
Superior laryngeal nerve injury	0	0	
Lymphatic ducts injured			
chyle leakage(n)	1	1	>0.05

Abbreviations: PTH, parathyroid hormone; RLN, recurrent laryngeal nerve.

No injury to superior laryngeal nerve and recurrent laryngeal nerve occurred. Only one patient (in Focus group) presented temporary recurrent laryngeal nerve paralysis. Intraoperative bleeding was not significant in any patient, and no neck hematoma, seroma, wound infection, or postoperative bleeding was observed. There were neither blood transfusions nor postoperative definitive sequelae. The mean postoperative hospital stay was reduced in the Focus group (*P *< 0.05). The rate of parathyroid gland autotransplantation was not statistically significant between Focus group (27.5%) and classic group (29.6%).

Two cases with positive lymph node dissection had postoperative chyle leakage. The maximal production of chylous fistula was about 1500 ml/d. Then they were subjected to high negative pressure drainage and local pressure dressing. Sandostatain and reasonable dietry control were applied in two patients. Chyle leakages stopped within 2 weeks.

## Discussion

The thyroid gland has an extensive vascular network, thus meticulous hemostasis is essential to ensure a dry surgical field and avoid inadvertent damage to adjacent vital structure. Although total thyroidectomy is one of the most common surgical procedures, whether it is the safest, most efficient and cost-effective way to achieve hemostasis is debated [9]. Means to prevent and control intra- or postoperative bleeding therefore remain a topic of utmost importance. In conventional surgery, electrocoagulation and suture ligation are used for haemostasis. The development of ultrasonic instruments in the early 1990s has provided an alternative to other methods of controlling blood vessels(e.g., Ligasure precise, lasers, clips, and staples)[10,11]. Despite their safety and effectiveness in thyroid surgery, the previous harmonic scalpel instruments are considered large and cumbersome by some surgeons. The new harmonic scalpel(Focus) has been made available since 2008. Recently, the Harmonic Focus has been used as an alternative to conventional hand-tied ligation for hemostasis in thyroid surgery[12-16]. The Harmonic Focus gave surgeons the versatility to perform several important functions(e.g., to dissect, cut, coagulate and grasp) without the need to exchange instruments. The device divides tissue by using 55.5 kHz ultrasonic energy transmitted between the instrument blades. This mechanical action disrupts protein hydrogen bonds within the tissue, and functions at a relatively low temperature (between 50°C and 100°C) to cause a lesser tissue injury compared with electrocautery (the Harmonic Focus causes lateral thermal injury 1.5 mm wide, approximately half of that caused by mono-polar systems).

Our data show that the Harmonic Focus allow a one third time saving vs classic hemostasis. Furthermore, the use of the Harmonic Focus would allow reduced traction and reduced manipulation of the thyroid, particularly when dividing the upper pedicle and transecting the superior pole, as claimed by Miccoli et al [17,18]. The reduced tissue injury and the better hemostasis are confirmed by the statistically significant reduction of drainage volume for patients in the Focus group. That reduction draws attention to the great sealing capacity of this device. After using the Harmonic Focus, some investigators reached similar conclusions regarding postoperative drainage, whereas others did not [19-23]. The Harmonic Focus proved to be safe, with good control of the blood vessels and no bleeding after surgery. At our institute, we used the Harmonic Focus exclusively for hemostasis in thyroid surgery, scarcely any ties.

Finally, our data demonstrate that the complication rate in the Focus group might not be significantly reduced. Incidences of definitive hypoparathyroidism, superior laryngeal nerve and recurrent nerve palsy were also not statistically significant. Intraoperative chyle leakage was not found in any patient, although postoperative leakage was detected in two patients. Postoperative lymphatic vessel leak or chylous fistula can be avoided by careful neck dissection in at-risk area during operation. Proper postoperative conservative management is of great help when leakage occurred. Careful dissection techniques using ultrasonic scalpels may be useful for preventing the occurrence of chyle leakage [24].

On the other hand, a major criticism of the Harmonic Focus is its cost [25-27]: the Harmonic Focus is disposable, expensive, yet must be considered an additional cost in the diagnosis related group's Medicare hospital payment system. However, when the reduced operating time and length of stay are considered, the device actually might be cost-effective. The use of the Harmonic Focus in multinodular goiter surgery allows for a significant reduction in the length of the procedure with a comparable cost. This represents a refinement of our current technique, with decreased anesthesia and operating time, and has clear economic impacts. But unfortunately, the Harmonic Focus is very expensive(¥7200 in China). The use of the harmonic Focus reduces surgical time, but increases the cost of the surgery. It is our belief that by including in the absolute cost the time saved and the reduction in human resources needed, the use of the Harmonic Focus would prove to be economic.

## Conclusions

The Harmonic Focus makes it fast and easy to perform a total thyroidectomy plus level III-IV and VI dissection without bleeding. The main advantage of this technique is that it simplifies the surgical procedure and eliminates the need to have repetitive "clip, cut and tie" maneuvers, while achieving efficient hemostasis. We have demonstrated that the Harmonic Focus is a new ultrasonic device in thyroid surgery, and offering great capabilities for delicate tissue grasping and dissection.

## Competing interests

The authors declare that they have no competing interests. This study was not sponsored by any company.

## Authors' contributions

QH, DZ and LZ had operated cases and analyzed all data. PZ, JC and ZL did the assistant of the operation. All authors read and approved the final manuscript.
